# Cooperation is related to dispersal patterns in Sino-Tibetan populations

**DOI:** 10.1038/ncomms9693

**Published:** 2015-10-19

**Authors:** Jia-Jia Wu, Ting Ji, Qiao-Qiao He, Juan Du, Ruth Mace

**Affiliations:** 1 Human Evolutionary Ecology Group, Dept. of Anthropology, UCL, 14 Taviton St, London WC1H 0BW, UK; 2 Theoretical Ecology Group, Key Laboratory of Animal Ecology and Conservation Biology, Institute of Zoology, Chinese Academy of Sciences, 1 Beichen West Road, Chaoyang District, Beijing 100101, PRC

## Abstract

There is growing recognition in both evolutionary biology and anthropology that dispersal is key to establishing patterns of cooperation. However, some models predict that cooperation is more likely to evolve in low dispersal (viscous) populations, while others predict that local competition for resources inhibits cooperation. Sex-biased dispersal and extra-pair mating may also have an effect. Using economic games in Sino-Tibetan populations with strikingly different dispersal patterns, we measure cooperation in 36 villages in southwestern China; we test whether social structure is associated with cooperative behaviour toward those in the neighbourhood. We find that social organization is associated with levels of cooperation in public goods and dictator games and a resource dilemma; people are less cooperative towards other villagers in communities where dispersal by both sexes is low. This supports the view that dispersal for marriage played an important role in the evolution of large-scale cooperation in human society.

## Abstract (web summary)

Dispersal is key to establishing patterns of cooperation. Here, the authors show that social organization is associated with levels of cooperation in Sino-Tibetan populations with strikingly different dispersal patterns.

## Graphical abstract

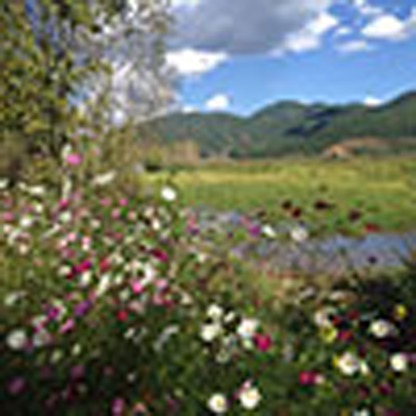

Cooperation beyond the family in human populations remains an evolutionary puzzle. Kin selection and inclusive fitness theory predict that natural selection favours cooperation in viscous populations as explained by Hamilton 1964 and developed by others in later theoretical models^1^,^2^,^3^,^4^,^5^,^6^, as neighbours will be more highly related to each other in populations with low dispersal. However including demographic and life history features of a population into the inclusive fitness models generates a contrasting prediction as low dispersal rate could increase local competition for reproductive resources between neighbours, and thereby inhibit the evolution of cooperation^7^,^8^,^9^,^10^,^11^. The determinants of cooperation within the family and within the neighbourhood may differ^12^. If competition happens locally, it can outweigh the effect of the high relatedness, so dispersal could promote cooperation among neighbours. As migrants are less related to their neighbours, they may be selected to be less cooperative than those who have not dispersed^9^. Asymmetries in relatedness can arise from sex-biased dispersal patterns resulting in differences in how those of different ages and sexes cooperate within in a population^13^. Furthermore, recent models predict that extra-pair mating can also have an effect on the evolution of cooperative breeding^14^ and on the level of cooperation between males in the neighbourhood^15^.

Human kinship structures are a traditional focus for anthropology, as humans show highly divergent norms of post-marital residence across cultures, sometimes even within the same region. Female-biased dispersal at marriage is the most common, resulting in patrilocal post-marital residence, whereas male-biased dispersal results in matrilocal residence. Non-dispersal of either sex results in a very rare kind of matrilineal system where residence is described as duolocal^16^. Female-biased dispersal and patrilineal descent tend to be associated with resources that males can monopolize to attract females^17^. In contrast, matrilineal descent systems tend to be associated with matrilocal or duolocal residence, horticulture and a lack of wealth such as livestock^18^. Female-biased dispersal generates different age-associated relatedness to one's group, which has been argued to influence age- and sex-biased patterns of cooperation and conflict^19^. Marriage links, arising due to pair-bonding and the exchange of females between clans, have also been hypothesized to play an important role in human cooperation beyond the immediate family^20^,^21^. These latter predictions are somewhat similar to those stemming from evolutionary theoretical models of dispersal increasing cooperation.

In southwest China, the matrilineal groups we study (the Mosuo, the Zhaba and a matrilineal population of Pumi) show a rare ‘duolocal' system where neither sex disperses. Both sexes remain in their multigenerational, natal household, often for life^22^,^23^,^24^, and men visit their wives or girlfriends only at night. In these duolocal populations, male parental investment is low and it is normal for females to raise children with their co-resident kin and without a co-resident husband. Extra-pair mating in these communities is thought to be higher than in the more restricted monogamous and/or patrilineal populations in the region such as the Han, Yi and patrilineal Pumi. The Amdo and Khampa are considered to be patrilocal but have looser marriage bonds and intermediate female dispersal rates (with bride and groom often from the same natal village); they have relatively high levels of unmarried mothers compared with patrilocal groups, who are usually living in their natal village, and could also have intermediate levels of extra-pair paternity. The co-existence of societies with no dispersal for marriage, within the same region, same language family, and following broadly similar agro-pastoral subsistence strategies, as patrilocal cultures with high and intermediate levels of female dispersal, provides a unique opportunity to examine the effects of dispersal patterns on human cooperation in a real world setting.

Here we report the results of a large, naturalistic and quasi-experimental study using economic games played by 720 people living in 36 villages, varying in their social organization. We can categorize each village as being a community with either high, intermediate or low female dispersal. Villages are natural communities in which individuals interact repeatedly and have the potential to cooperate with farming, childcare and in any other domain of human life. We used multiple games: a dictator game (DG), a one-shot public goods game (PGG) and a resource dilemma game (RDG)^25^,^26^,^27^,^28^, and examined kinship and other determinants of variation in cooperative behaviour within and between communities. In these games, more cooperation is defined as giving more in the DG, contributing more in the PGG and taking less tea in the RDG. Generated from the predictions of theoretical models, we tested the following hypotheses: (a) lower dispersal communities have both a higher density of kin and a higher possibility of extra-pair mating, thus will show a higher level of dictator giving, public goods contribution and a lower level of resource taking; (b) alternatively, there is competition for reproductive resources between kin, so lower dispersal communities will show a lower level of dictator giving, public goods contribution and higher resource taking; (c) migrants are less cooperative in the games; (d) dispersal norms interacts with sex and age. We find that social organization is associated with levels of cooperation in both public goods and dictator games and a resource dilemma; people are less cooperative towards the other villagers in communities where dispersal by both sexes is low.

## Results

### Differences in rates of female dispersal between groups

**Figure 1:**
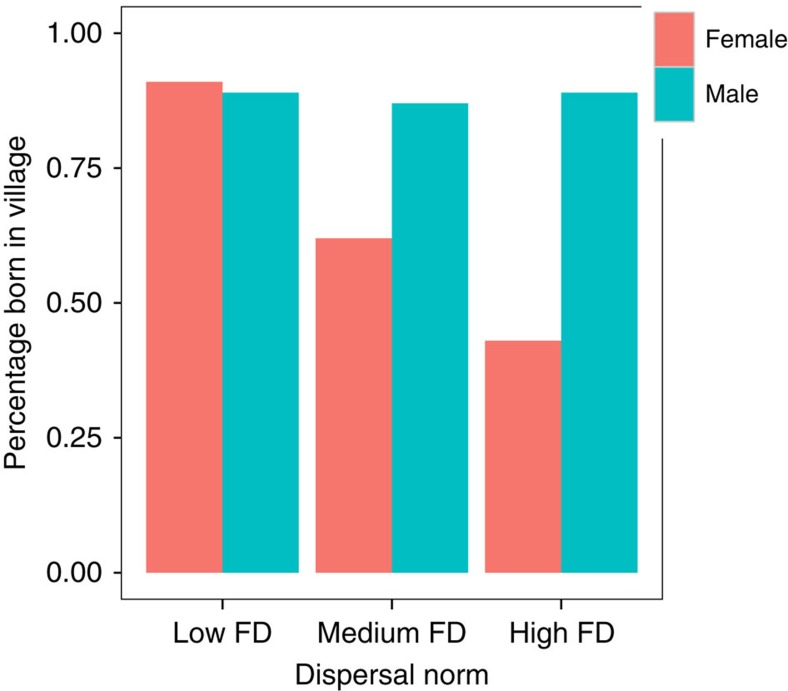
Proportion of women and men born in the village they are living in for communities of different dispersal norms. A total 9% of women in low FD (female dispersal) villages were born outside of the village, whereas 57% of women in high FD villages moved to another. The proportion of males dispersing is low in all communities. The sample size was, for low FD communities, *N*=280, 53% male; for high FD communities *N*=300, 51% male; and for medium FD communities, *N*=140, 57% male.

Less than 10% of women in villages of low FD (low female dispersal) were born outside of the village, whereas 57% of women in villages of high FD (high female dispersal) had moved in from another village, with medium FD (intermediate female dispersal) villages in between these values (see Fig. 1). This makes adult average relatedness to others in the neighbourhood highest in low FD and lowest in high FD populations (Supplementary Fig. 1). Males do not normally disperse in any of these groups (Fig. 1 and Supplementary Fig. 1).

### Effects of sex and age and close kin in the game

Using multilevel models, we investigate effects on cooperation of village level variables such as dispersal norm and sex ratio in the game (Supplementary Tables 1 and 2), while controlling for the effects of individual level variables such as sex, age and number of close kin in the game. Assuming selfishness, the rational strategy or Nash equilibrium in a one-shot anonymous game is to give nothing in the DG, contribute nothing in the PGG and extracting all resources in the RDG, optimizing immediate gains. But very few players follow those selfish strategies; nearly all donate something and very few take all the tea. It is known that in other games where players can make donations to named individuals, players tend to favour close kin^29^, so we control for the number of close kin in the games in case that influenced donations to the group. At the individual level (see Table 1), the number of close kin playing in the same game has a positive effect on giving in the DG (one more close kin attending the game makes people to give 0.5 Yuan more to the recipient), and being born in the village significantly increases contributions in the PGG, suggesting close relatives do increase cooperation. However, a spouse attending the same game has no effect. Men are more cooperative than women in all games; significantly so for public goods contribution and resource taken in the RDG, and marginally significant for dictator giving. This sex difference is not observed in many lab-based dictator game experiments on western undergraduate subjects and in the lab^30^,^31^. It is interesting to note that the DG donation, which is sometimes described as altruism rather than cooperation, is the game that is most responsive to close kin in the game.

### Effects of dispersal norms and sex ratio in the game

**Figure 2:**
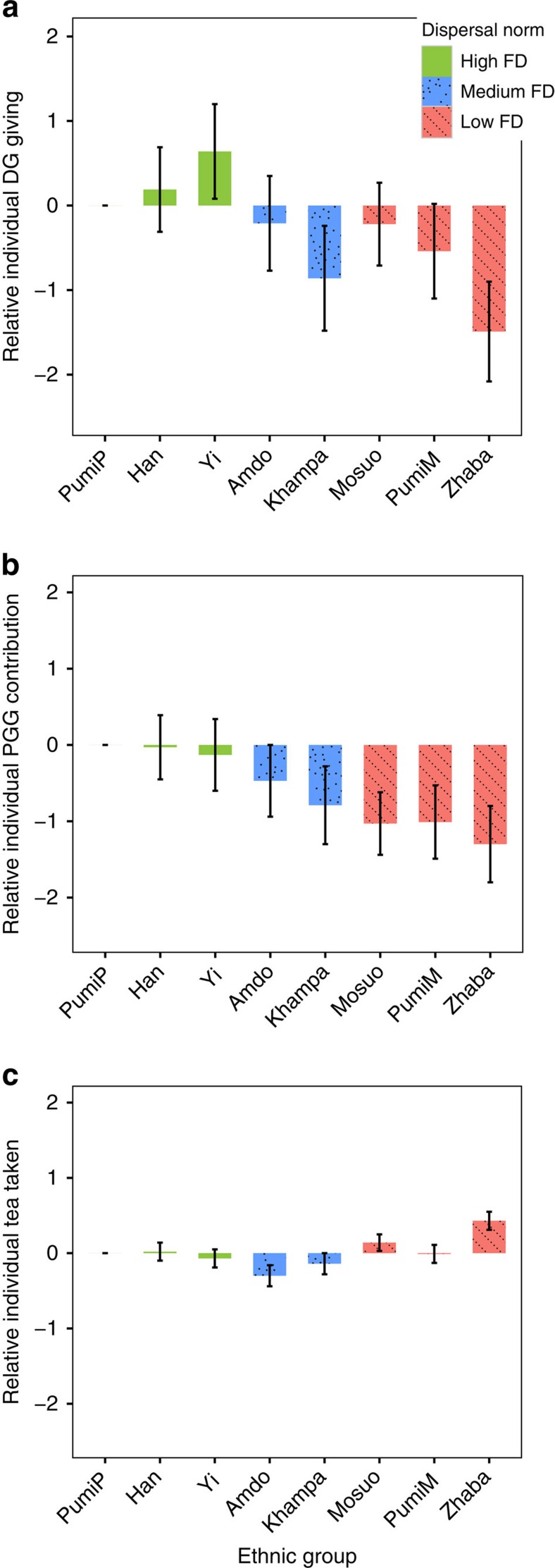
Relative average individual donations in the DG and the PGG and tea taken in the RDG for different ethnic groups. Using linear regression for DG (*N*=360) (**a**) and PGG (*N*=720) (**b**) and Poisson regression for RDG (*N*=561) (**c**) controlled for age and sex, with patrilocal-Pumi (PumiP) as reference. Each bar represents the value relative to the patrilocal-Pumi with age and sex controlled for. Green bars, high FD; blue bars filled with dots, medium FD; red bars filled with backslash, low FD. Error bars indicate the standard error from the mean. Note that high values in the RDG game denote selfishness (taking more tea from the public pot), whereas low values indicate selfishness (less dictator giving and public goods contribution) in the PGG and DG.

At the village level, low FD has a significant negative association with dictator game giving and public goods contribution (Table 1). Figure 2 and Supplementary Table 3 shows relative average game outcome of participants by ethnic group, controlling for sex and age (with Supplementary Fig. 2 and Supplementary Table 4 showing raw data and uncontrolled effects). People in low FD communities (red) offer significantly less than those in high FD communities (green), and medium FD communities are intermediate in both economic games. Low FD communities are also more selfish in terms of taking more tea. There are no significant interactions between dispersal norm and age, although the dispersal norm difference appears to derive more from men's behaviour rather than women's (see Supplementary Note 1 and Supplementary Tables 5 and 6). When there are more men in the game, people give less to the public goods game and take more tea from the public pool, over and above the individual effect of sex. Even though games are anonymous, this is consistent with men behaving as if they are signalling their cooperativeness (and possibly also their wealth) with a higher level of donation, especially to women in high FD populations where the women are less related to them.

We also examine whether wealth is associated with cooperative behaviour in a sub-sample of villages where we have complete wealth rankings, and wealth rank in the village seems not to be important (See Supplementary Table 7). Having an occupation (other than farmer/herder) is associated with larger contributions in the PGG (Table 1 and Supplementary Table 7), but this effect does not remove the significant negative effect of low FD.

In summary, a lower dispersal rate is associated with a lower level of cooperation in economic games. These results support the prediction that low female dispersal increases local competition for resources^7^,^10^,^13^, leading to less cooperation in the neighbourhood. They do not support the prediction that low dispersal resulting in high relatedness in the population promotes cooperation. Given that mating patterns are generally less restrictive in matrilineal systems and that these are also the low dispersing groups, there is no support for Eliassen and Jørgensen's hypothesis^15^ that a social system with more extra-pair mating promotes cooperation between males.

## Discussion

We find that communities with low dispersal show less cooperation with others in the neighbourhood. Previously, we found that duolocal females experience reproductive competition at the household level, as those that move out of group households into neolocal households have higher fertility^23^. Traditionally, fertility in the duolocal Mosuo has been quite low^32^. Marriage bonds are traditionally weak or non-existent in duolocal communities. It is possible that duolocal people are more oriented toward cooperation within the household in this unusual system of large, highly related households, as some of the ethnographic literature suggests^33^. Wider social networks outside the household, created partly by linking affines through marriage, may be the basis of large-scale cooperation and these wider networks may be more important in patrilineal systems, and for males in general. Thus, these unusual duolocal societies, in which dispersal for marriage does not normally occur, provide a unique opportunity to show how low dispersal inhibits large-scale cooperation.

Kin effects and other effects may be sensitive to the precise ecological context of the cooperative behaviour. Some other studies have addressed the difference in cooperative behaviour between an individual matrilineal and patrilineal group. One study using a dictator game (with single sex groups that were also a bit smaller) in this area found matrilineal Mosuo men not only give more than women, as we find, but also more than patrilineal Yi^34^. One study using a public goods game in India found matrilineal Khasi contribute more than patriarchal Assam^35^, but another study using an Ultimatum game in Africa demonstrated that matrilineal Pimbwe give less than patrilineal Kukama^36^. These studies did not report whether or not close kin were playing in the game. A test of cooperation using economic games in a real world setting in India found that a high number of female kin in the village was associated with lower contributions in a PGG^28^. This also suggests that dispersal might enhance cooperation but it is not tested explicitly. These studies only used one group to represent social organization and did not consider dispersal patterns explicitly. Some lab studies, often using western college students as subjects, find no sex effect or find females tend to give more than males^30^,^31^, but such studies are not really informative on the effects of ecological context. Thus differences might arise due to differences in experimental and subjects' characteristics, such as differences in age, family members and sex ratio in the group playing, which variables were controlled for in the analyses, ecological context and smaller sample sizes.

Although many economic games have been criticized as subject to framing, lack of external validity, lack of context and other difficulties in interpretation^37^,^38^,^39^, they can provide a controlled means of comparing cooperative relationships in real communities, and as such can potentially reveal different social norms in different social organizations^40^,^41^,^42^. Our study design minimizes confounds by comparing many communities within one language family and within a relatively small geographical region, with similar ecology, environment and social conventions, using the same research team with identical experimental procedure and protocol, and providing enough replicates at individual, as well as community level to enable multilevel analysis to investigate sources of variation both within and between communities with different social organizations. The results of our study reveal that, although players may strategically adjust their cooperative level upward if close kin are in the game, in communities with low dispersal rates, we see less rather than more cooperative behaviour beyond the immediate family. This supports the broader notion that dispersal for marriage may be a key factor in establishing large-scale cooperation in human societies.

## Methods

### Population studied

We conducted this research in Lugu Lake, Dawu, Sichuan Province and Maqu, Gansu Province, Southwest China in 2013 and 2014. We used a quasi-experimental design, making use of strikingly different dispersal rates arising from different kinship patterns in seven ethnic populations: patrilocal groups with high female dispersal, where most female move to their husbands' village after marriage; duolocal groups with low female dispersal, where both men and women stay in their natal house throughout their life (the details of duolocal residence are described elsewhere^22^,^23^,^43^); ambilocal groups with intermediate female dispersal, where husband and wife live together but the bride is often also from the same natal village (and/or returns to her natal village after divorce, which is quite common in these communities). Each dispersal norm included villages from two or three ethnic group populations: high FD groups were agricultural Yi, Han and patrilocal Pumi living around Lugu Lake on the border of Yunnan and Sichuan Province; medium FD groups were Amdo pastoralists living in Maqu, Gansu Province and agricultural Khampa in Dawu, Sichuan Province; low FD groups were agro-pastoral Zhaba living in Dawu, Sichuan Province^24^, agricultural Mosuo and a duolocal population of Pumi in Lugu Lake^16^.

### Games

We carried out games in 36 different villages, each game involving 20 residents who would potentially interact with each other in the daily life of that village. At each village, we gathered the 20 adult participants randomly from neighbourhood. The sex ratio averaged 50:50 but varied between villages due to the availability of people at each game session, enabling us to examine whether behaviour correlates with sex ratio in the game. Once the player group was assembled, we gave an explanation of the procedure of the games in front of all the participants. Then each individual was told the instructions again and played the games in private, without knowing which of the 20 individuals they were matched with in the PGG or DG. When the session finished, each participant received an envelope with all his or her earnings in cash from the games, on average 32 Yuan for 1 h, which is about half of 1 day's wage in that area.

A game session consisted of a standard anonymous dictator game^25^, with half the participants dictators and half recipients, with the dictators asked to divide 10 Yuan between themselves and an anonymous recipient; a standard anonymous one-shot public goods game^26^,^27^, where the 20 participants were randomly divided into five anonymous groups of four, with a multiplier of 2 added for the contributions to the pot; and a resource dilemma game^28^ where each participant withdrew tea packets from a public pool, unless the pool had already been exhausted by earlier players (see Supplementary Methods for protocols, which were similar to those used in ref. 28). The essential difference between these games is that the PGG involves some expectation of how cooperatively others will behave in contributing to the public pool, as their contribution will influence the return, whereas the dictator game is just a donation (with no expectation of any returns from the recipient), and the resource dilemma game is a decision regarding how much to deplete a common resource.

### Demographic data

Demographic data were collected after the games through a questionnaire that included basic individual information such as name, sex, age, animal sign, ethnic group, marital status, post-marital residence, birth place, number of adults and children in the household and occupation; and kinship information on number of close kin (including parents, sibling and adult children) alive and living in the village, and the number of close kin and spouse playing in the game session.

### Statistics

We used multilevel models to analyse the game outcomes at the village and individual levels in our nested data (720 individuals within 36 villages) for dictator game, public goods game and resource dilemma game separately. We estimated linear models with a normal error distribution in the DG and PGG, and generalized linear models with a Poisson error distribution and a log link function in the RDG. The response variables in the models were the dictator giving in the DG, public goods contribution in the PGG and the tea taken in the RDG. The full model had individual-level predictor domain, including sex and age as control, occupation, birth place and kin and spouse in the game as kinship predictors, and village-level predictor domain, including dispersal norm and sex ratio in each village, while village was treated as a random effect to control for the variances in different villages. Age was standardized to a normal score with a mean of zero and unit variance. Tea fair share was included as an individual variable in the models of the RDG to control for the variance of tea depleting rate. The analysis was carried out using the statistical computation system R 3.0.3 (ref. 44), including lme4 and LmerTest Packages, and *P* value was estimated by using Satterthwaite approximations^45^. Multilevel models are recommended for estimating standard errors in samples of cluster numbers in the range used in this study^46^,^47^. All the models controlled for age and sex. We compared the control and full models by using an information criteria-based approach (Supplementary Table 8)^48^,^49^.

The research was approved by the Research Ethics Committee at University College London and the Chinese Academy of Sciences, Beijing, and informed consent obtained from all the participants.

## Additional information

**How to cite this article:** Wu, J.-J. *et al*. Cooperation is related to dispersal patterns in Sino-Tibetan populations. *Nat. Commun.* 6:8693 doi: 10.1038/ncomms9693 (2015).

## Supplementary Material

Supplementary Information Supplementary Figures 1-2, Supplementary Tables 1-8, Supplementary Note 1, Supplementary Methods and Supplementary Reference

## Figures and Tables

**Table 1:** Estimates of control and full multilevel models for dictator game (DG) and public goods game (PGG, linear) and resource dilemma game (RDG, Poisson).

Estimate	Dictator game (*N*=358)				Public goods game (*N*=718)				Resource dilemma game (*N*=560)			
	Control		Full		Control		Full		Control		Full	
	Est	Se	Est	Se	Est	Se	Est	Se	Est	Se	Est	Se
Intercept	5.04	0.25	**5.31**	0.56	5.63	0.21	**6.47**	0.41	0.62	0.08	−**0.05**	0.29
Fixed effect												
Age	−0.07	0.12	−0.11	0.12	−0.14	0.11	−0.12	0.11	−0.01	0.03	−0.02	0.03
Sex	**0.57**	**0.27**	0.58	0.3	**1.09**	**0.23**	**0.94**	**0.26**	−**0.18**	**0.06**	−**0.16**	**0.07**
TeaFS	*				*				**0.005**	**0.002**	**0.005**	**0.002**
Occupation			−0.09	0.34			**0.6**	**0.3**			−0.11	0.09
Birth place			−0.04	0.35			**0.55**	**0.29**			0.01	0.09
Close kin in game			**0.5**	**0.18**			0.09	0.15			0.03	0.04
Partner in game			0.25	0.37			0.18	0.31			0.02	0.08
Low FD			−**0.9**	**0.44**			−**0.97**	**0.32**			0.16	0.1
medium FD			−0.87	0.54			−0.43	0.39			−0.23	0.12
Sex ratio			0.04	0.89			−**1.75**	**0.65**			**1.52**	**0.56**
Random effect												
Village	1.061	1.03	0.802	0.895	0.622	0.789	0.281	0.530	0.064	0.253	0.031	0.176
Individual	5.426	2.329	5.317	2.306	8.138	2.853	8.054	2.838				
VPC	16.4%		13.1%		7.1%		3.37%					

Linear multilevel models were used in the DG and PGG and generalized linear multilevel models (Poisson) were used in the RDG, with response variables dictator giving in the DG, public goods contribution in the PGG and tea taken in the RDG. The fixed effect predictors used in the models are sex (male=1, female=0), age (standardized age), birth place (in village=1, outside=0), close kin in the game (number of *r*=0.5 kin attending the same game), partner in the game (partner in=1, not=0), occupation (having job other than farmer/ herder=1, not =0), and fair share of tea (for RDG game) as individual level variables, sex ratio (proportion of men playing in the game) and dispersal norm (High FD as reference) as village level variables. Estimates in bold are significant at *P*<0.05, with *P* value calculated by using Satterthwaite approximations to estimate degrees of freedom (merModLmerTest package).

^*^For Dictator game and public goods game, the variable Tea fair share was not included in the analysis, so the cells for the variable are empty, because only in the RDG does the fair share change as previous players deplete the common pool resource.
