# Supplementary material for: Cooperation is related to dispersal patterns in Sino-Tibetan populations
Source: Nat Commun. 2015 Oct 19;6:8693. doi: 10.1038/ncomms9693 (PMC4667691; doi:10.1038/ncomms9693)
Supplement: Supplementary Information — Supplementary Figures 1-2, Supplementary Tables 1-8, Supplementary Note 1, Supplementary Methods and Supplementary Reference [file ncomms9693-s1.pdf]

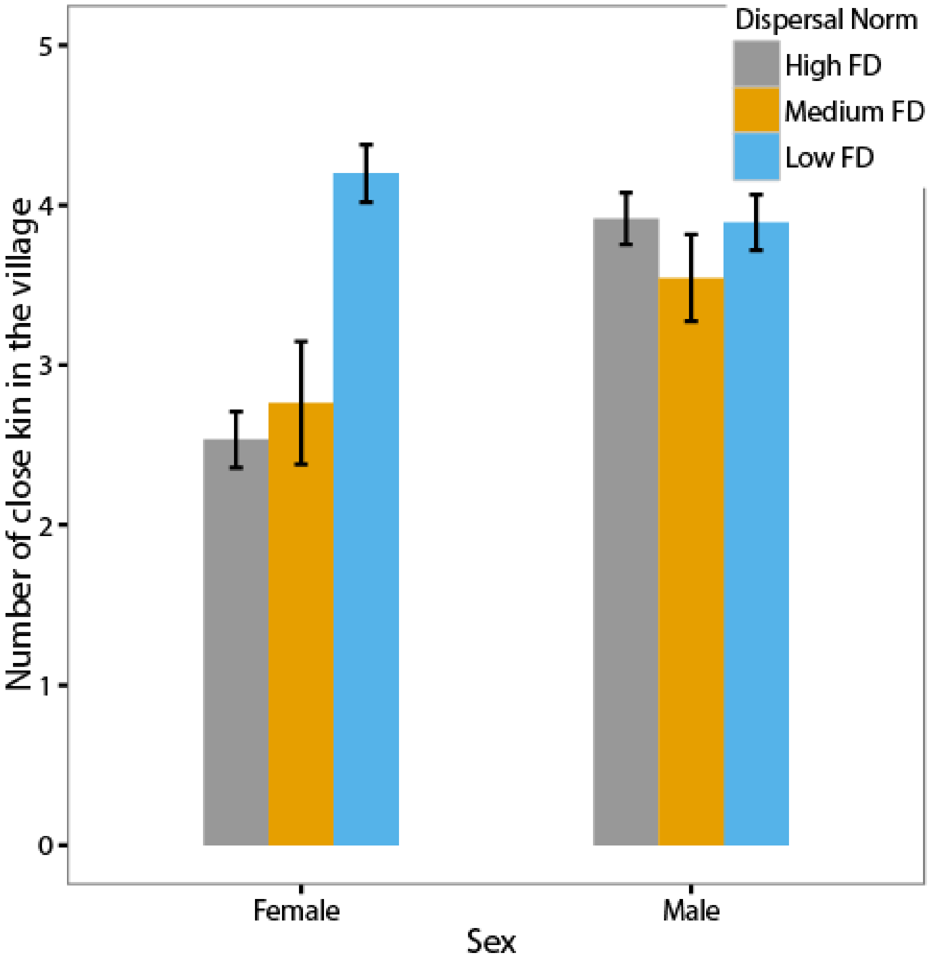

Supplementary Figure 1 Average number of close kin in village for men and women in low and medium and high FD communities respectively. Low FD women have more close kin in the village than high and medium FD women (both $p<0.001$), no difference between high and medium FD women ($p=0.79$), ($N=338$, TukeyHSD multiple comparison). There's no difference of close kin number between men in different communities, ($N=382$, TukeyHSD multiple comparison, $p=0.99$, 0.42, 0.47). Grey bars, high FD; orange bars, medium FD; blue bars, low FD. Error bars represent mean ± SE

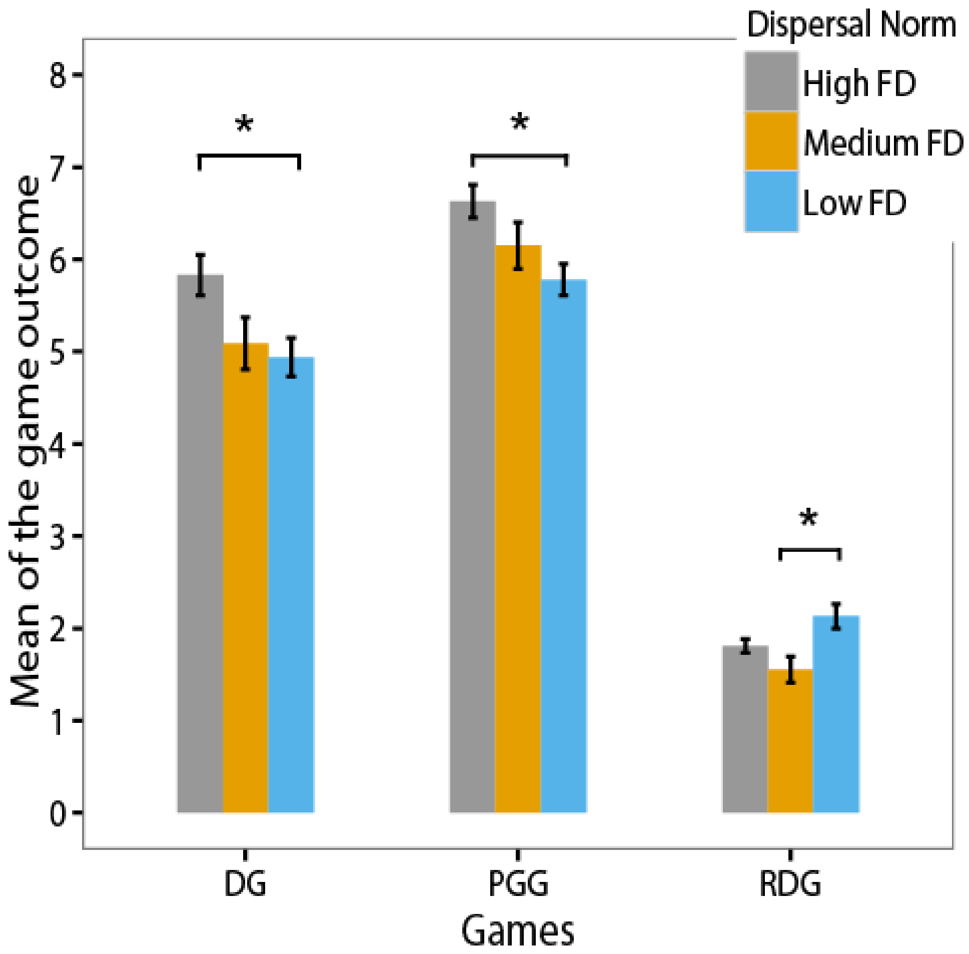

Supplementary Figure 2 Average individual donations in the DG and the PGG and tea taken in the RDG for different dispersal norms. There were significant differences between communities with low, medium high female dispersal norms in all three games (Dictator Game (DG) N=360 [F(2,357)=4.868, p=0.0082], Public Goods Game (PGG) N=720 [F(2,717)=6.013, p=0.00257], Resource Dilemma Game (RDG) N=561 [F(2,558)=5.827, p=0.00313], one-way ANOVA). Post hoc comparisons using the Tukey-Kramer test indicated that the average dictator giving for high FD (M=5.83, SD=2.69) was significantly different than that for low FD (M=4.94, SD=2.49) (95% CI[-1.61,-0.17]), average public goods contribution for high FD (M=6.63, SD=3.09) was significantly different from that for low FD (M=5.78, SD=2.82) (95% CI[-1.43,-0.28]), and average tea taken for medium FD (M=1.55, SD=1.54) was significantly different from that for low FD (M=2.13, SD=1.83) (95% CI[-1.04,-0.13]). Grey bars, high FD; orange bars, medium FD; blue bars, low FD. Error bars represent standard error, and asterisk shows the significant difference between groups.

Supplementary Table 1 Null control and full multilevel models for the DG and the PGG (Linear) and the RDG (Poisson)

Game	Models	Fixed effect	Random effect	Numbers of observation/ game	AIC	df. residual	logLik	Delta AIC
DG	Null		village	360/36	1680.4	357	-837.2	
	Control	Age+ sex	village	360/36	1679.4	355	-834.7	1
	Full	Control+ OP+ BP+ CK+ PT + DN+ SR	village	358/36 [a]	1671.1	346	-823.5	8.3
PGG	Null		village	720/36	3615.5	717	-1804.8	
	Control	Age+ sex	village	720/36	3596.2	715	-1793.1	19.3
	Full	Control+ OP+ BP+ CK+ PT + DN+ SR	village	718/36 [a]	3578.5	706	-1777.2	17.7
RDG	Null		village	561/29	1834.4	559	-915.2	
	Control	Age+ sex	village	561/29	1825.3	556	-909.9	9.1
	Full	Control+ OP+ BP+ CK+ PT + DN+ SR + FS	village	560/29 [a]	1821.4	548	-898.7	3.9

OP occupation, BP birth place, CK close kin in game, PT partner in game, DN dispersal norm, SR sex ratio, FS fair share of tea

a Among all participants, half of them played the dictator game (N=360, two missing because of no occupation mentioned), all of them played the public goods game (N=720, two missing because of no occupation mentioned), not all the villages played the Resource Dilemma game. Within those groups played the Resource Dilemma game, not all participants got tea because when the tea was gone, the game finished no matter how many participants left.

Supplementary Table 2 Individual level variables in the models

N=720	High FD	Medium FD	Low FD	in total
Participants	300	140	280	720
male%	51.3%	57.1%	52.9%	53.1%
born in village%	66.7%	76.4%	89.6%	77.5%
occupation%	25.1%	5.0%	25.1%	21.2%
Partner in game%	16.7%	30.0%	7.9%	15.8%
Close kin in game%	38%	36.4%	36.8%	37.2%
Single%	1.3%	6.4%	14%	7.4%
Average age	42.41 ±13.85	39.04 ±13.26	41.6 ±12.94	41.44 ± 13.43

Supplementary Table 3 Average relative individual donation in the DG and PGG (Linear) and tea taken in the RDG for different ethnic groups

Estimate	DG ± SE (N=360)	PGG ± SE (N=720)	RDG ± SE (N=561)
(Intercept)	**5.2519 ± 0.4125**	**6.31294 ± 0.34713**	**0.69352 ± 0.08721**
Sex	**0.5946 ± 0.2696**	**0.85184 ± 0.2219**	**-0.19047 ± 0.06276**
Age	-0.1033 ± 0.1305	-0.12585 ± 0.11089	-0.01326 ± 0.03126
Han	0.1892 ± 0.5019	-0.02886 ± 0.41565	0.02046 ± 0.11613
Yi	0.636 ± 0.561	-0.13369 ± 0.46679	-0.07218 ± 0.11922
Amdo	-0.2061 ± 0.5638	-0.47487 ± 0.46819	**-0.30473 ± 0.13815**
Khampa	-0.8624 ± 0.6194	-0.79241 ± 0.51418	-0.13502 ± 0.13572
Mosuo	-0.2202 ± 0.4945	**-1.02534 ± 0.41057**	0.1415 ± 0.11176
PumiM	-0.5404 ± 0.5643	**-1.00723 ± 0.47942**	-0.01153 ± 0.12035
Zhaba	**-1.4904 ± 0.5976**	**-1.30208 ± 0.49562**	**0.43205 ± 0.12111**

Estimates from single linear regression for the DG giving, PGG contribution, and single Poisson generalized linear regression for tea taken, as a function of sex (reference=female), age (standardized age) and ethnic groups (patrilocal-Pumi as reference) in the DG, PGG and RDG separately. Estimates in bold are significant at 0.05 level.

Supplementary Table 4 Descriptive statistics of three Games.

Game	Dispersal Norm	N	min	1st Qu	Median	Mean	3rd Qu	Max
Dictator Game	total	360	0	5	5	5.336	7	10
	Low FD	140	0	5	5	5.827	8	10
	Medium FD	70	0	5	5	5.086	5.75	10
	High FD	150	0	4	5	4.936	6	10
Public Goods Game	total	720	0	5	6	6.204	9	10
	Low FD	280	0	5	7	6.63	10	10
	Medium FD	140	0	4	6	6.15	9	10
	High FD	300	0	4	5	5.775	8	10
Resource Dilemma	total	561	0	1	1	1.87	2	11
Game	Low FD	202	0	1	2	1.812	2	6
	Medium FD	119	0	1	1	1.546	2	11
	High FD	240	0	1	2	2.129	2	10

Supplementary Table 5 The effect of age and dispersal norm interaction for females in the DG and the PGG (Linear) and the RDG (Poisson)

Female	DG (n=170)		PGG (n=336)		RDG (n=294)	
	Estimate	Std. Error	Estimate	Std. Error	Estimate	Std. Error
(Intercept)	**5.10773**	**0.60914**	**6.3929**	**0.474**	-0.20675	0.268673
Age	0.17673	0.34906	-0.11908	0.25902	-0.08138	0.068412
low FD	-0.15782	0.52268	-0.52677	0.40492	0.198304	0.105192
medium FD	0.16658	0.63381	0.13618	0.47186	-0.12475	0.120631
birth place	0.00121	0.51418	0.39355	0.40176	-0.03087	0.109173
sex ratio	-0.54351	1.25997	**-2.10644**	**1.01597**	**1.903444**	**0.519259**
close kin in game	0.37133	0.29355	0.0434	0.21849	0.041688	0.052592
spouse in game	0.30709	0.58096	0.23775	0.44959	-0.04674	0.111173
Occupation	-0.10412	0.87146	**1.80004**	**0.75079**	0.061792	0.183265
Tea fair share	[a]		[a]		-0.00174	0.002887
Age: low FD	-0.44866	0.46124	-0.09627	0.37952	0.02777	0.096714
Age: medium FD	0.21032	0.69615	-0.2868	0.46968	**0.277944**	**0.118308**

Bold estimates are significant at 0.05 level.

a, for Dictator game and public goods game, the variable Tea fair share was not included in the analysis, so the cells for the variable are empty, because only in the RDG does the fair share change as previous players deplete the common pool resource.

Supplementary Table 6 The effect of age and dispersal norm interaction for male in the DG and the PGG (Linear) and the RDG (Poisson)

Male	DG (n=188)		PGG (n=382)		RDG (n=266)	
	Estimate	Std. Error	Estimate	Std. Error	Estimate	Std. Error
(Intercept)	**6.11031**	**0.77451**	**7.4379**	**0.6927**	0.035352	0.352935
Age	0.11922	0.24332	-0.2676	0.2184	0.0114	0.063679
low FD	**-1.57934**	**0.39233**	**-1.2197**	**0.3383**	0.122341	0.108626
medium FD	**-1.39626**	**0.47671**	**-0.8886**	**0.4282**	-0.28334	0.145995
birth place	-0.20376	0.57607	0.772	0.4728	-0.04063	0.154628
sex ratio	0.21401	0.76547	**-1.6609**	**0.6696**	0.887556	0.614583
close kin in game	**0.68097**	**0.22694**	0.1826	0.1982	0.010288	0.062722
spouse in game	0.14184	0.51129	0.18	0.4429	0.040374	0.122445
Occupation	0.08956	0.39096	0.298	0.3372	-0.17065	0.109373
Tea fair share	[a]		[a]		**0.008343**	**0.003225**
Age: low FD	**-1.0891**	**0.36264**	0.2465	0.3257	-0.09233	0.100526
Age: medium FD	0.33725	0.41106	0.5968	0.3932	-0.06458	0.131867

Bold estimates are significant.

a, for Dictator game and public goods game, the variable Tea fair share was not included in the analysis, so the cells for the variable are empty, because only in the RDG does the fair share change as previous players deplete the common pool resource.

Supplementary Table 7 The effect of wealth on cooperative behaviour in a sub-sample of villages

Model Estm.	DG (N=115)		PGG (N=233)		RDG (N=95)	
	Estimate	SE	Estimate	SE	Estimate	SE
Intercept	**4.25035**	**0.52423**	**5.70778**	**0.43041**	**0.616013**	0.210716
Age	-0.33733	0.21334	-0.02467	0.18189	0.051484	0.073399
Sex	0.97292	0.55031	-0.08624	0.44977	-0.152944	0.222445
Low FD	-0.63303	0.48886	**-1.23736**	**0.42518**	[a]	
Medium FD	-1.07435	0.61333	-0.29309	0.53914	-0.485658	0.262854
Birth Place	0.6161	0.58863	**1.12259**	**0.44622**	-0.063401	0.215186
Close Kin In Game	**0.69461**	**0.29332**	0.16468	0.24634	0.032035	0.103402
Partner In Game	0.38555	0.68518	0.56895	0.5885	0.144989	0.186301
Poor-Wealth Rank	0.48331	0.62296	-0.32394	0.57343	-0.065564	0.281739
Rich-Wealth Rank	0.2169	0.43362	-0.18419	0.38372	-0.013301	0.186362
Occupation	-0.02714	0.51785	**0.92275**	**0.44859**	-0.223976	0.217608
Tea fair share	[b]		**[b]**		0.007175	0.005293

Estimates from single linear regression for the DG giving, PGG contribution, and single Poisson generalized linear regression for tea taken, as a function of wealth (medium as reference, data from population census), sex (reference=female), age (standardized age), dispersal norm (high FD as reference), birth place (outside of the village as reference), close kin in the game (number of r=0.5 kin attending the same game), partner in the game (partner not in the game as reference), occupation (being a farmer or herder as reference), and fair share of tea (only in RDG) in the DG, PGG and RDG separately. Estimates in bold are significant at 0.05 level.

a, for RDG game, the low FD participants are not included in population census data so that no wealth ranking data to put into the analysis.

b, for Dictator game and public goods game, the variable Tea fair share was not included in the analysis, so the cells for the variable are empty, because only in the RDG does the fair share change as previous players deplete the common pool resource.

Supplementary Table 8 Model comparison between multilevel regression and single level regression for null and full model

Models	DG			PGG			RDG		
	AIC	logLik	Delta AIC	AIC	logLik	Delta AIC	AIC	logLik	Delta AIC
Single level NULL	1704.121	-850.06		3625.819	-1810.91		1851.117	-924.558	
Multilevel NULL	1680.441	-837.221	23.68	3615.52	-1804.76	10.299	1834.38	-915.19	16.737
Single level FULL	1685.673	-831.837		3581.986	-1779.99		1826.763	-903.382	
Multilevel FULL	1671.088	-823.544	14.585	3578.486	-1777.24	3.5	1821.422	-898.711	5.341

Supplementary Note 1 Dispersal norm interactions with age and sex

Johnstone and Cant model[1] predicts that the dynamic of female's relatedness to the group may differ with age in communities with different disperse rate, where higher dispersal rate leading to increasing of relatedness to the group thus older women in such population should show more tendency to cooperate. To test this hypothesis, we conducted regression models including age and dispersal norm interaction for female and male separately. We found no interaction between age and dispersal norm in the DG (N=170) and PGG (N=336) for females and in the PGG (N=382) and RDG (N=266) for males, but in the RDG (N=294) medium FD females take more tea with age and in the DG (N=188) low FD males give less with increasing age (see Supplementary Table 5 and 6). This result does not support the prediction from the model that higher relatedness to the group when females are older makes them more cooperative.

Supplementary Methods Experimental protocols and procedure

Experimental procedure

We conducted this research in the Sino-Tibet region, Southwest China from June to July 2013 and September to October 2014, covering seven ethnic populations. These were agricultural Yi, Han, and a patrilocal population of Pumi living around Lugu Lake on the border of Yunnan and Sichuan Province, Amdo agropastoralists living in Maqu, Gansu Province, agricultural Khampa in Dawu, Sichuan Province, agro-pastoral Zhaba living in Dawu, Sichuan Province, agricultural Mosuo and a matrilineal duolocal population of Pumi in Lugu Lake.

The same investigators went to each village where mainly one of seven ethnic groups resides to conduct the experiments with the same experimental protocol. On arrival at each village, after informing the village through notices and word of mouth that we were playing economic games, we gathered a group of 20 volunteer participants for each game session (all adult local residents over 16 years old, roughly half of whom were women). Then we described the experimental protocols to the whole group of participants, including the experimental procedure and the instruction of the Dictator game and Public Goods game but not the Resource Dilemma game.

First, each participant took an envelope with the number of order of play, and played the game one by one in order in a separate room (or in the car of the investigators) without his/her decisions becoming public. When the participant came into the private area, we told him/her which game s/he was to play and explained the instruction again before s/he made any decisions. They played the DG first, then the PGG. If there were tea packets left, s/he then played the Resource Dilemma game. Other participants waited their turn and were told not to talk about the experiment. After each participant finished his/her games, s/he was asked to fill in a demographic questionnaire. Right after all the participants had finished all games, their earnings from the games were calculated by the investigators and they each received an envelope with all money they gained from the games and the show-up fee in cash. For an average one and a half hour game, each participant gained 32 Yuan (RMB) on average, which is about half of a single days wage for unskilled labor working in that region

Summary of instructions

First, each participant needs to sign the consent before the game began.

Second, each participant will get 10 Yuan (CNY) as a show-up fee and attend no more than one session.

Third, a session consists of two games. In the first game, the computer will randomly choose 10 of the participants as proposers, who will make a decision on distributing some money between himself/herself and a randomly chosen anonymous recipient in the remaining 10 participants cannot make any decision in this game Then 10 proposers will receive 10 Yuan each and decide how much to keep to themselves and how much to give to the anonymously paired recipient. The proposer can give any amount of money to the recipient between 0-10 and keep the rest for himself/herself.

In the second game, the total 20 participants are randomly and anonymously divided into 5 groups with four people in each group, and four people will make a decision on distributing some money between himself/herself and a public pot. Each participant will receive 10 Yuan and decide whether to contribute to the public pot. The participant can contribute any amount of money between 0-10 and keep the rest to himself/herself. After each participant makes their decision privately, the

public pot will be doubled and the total money split evenly between the four participants. Participants' total payoff will be the part they kept for themselves and the part gained from public pool.

After finishing the previous one or two games, the participant is asked to make a decision on a Resource Dilemma Game, if there are any tea packets left. Initially we bring 200 (x) packets of tea in total for 20 (y) participants to withdraw in each session, so 10 packets per participant is the starting fair share. During the game, we tell the participant the fair share but that the participants can take as much tea as they want and leave the room with those tea packets, which the other participants know nothing about. After the previous participant takes the tea packets s/he wants, we count how many tea packets are left (x), and divide that number by the number of the remaining participants including the current participant (y), to get his/her fair share (z=x/y); we then tell her/him that s/he can take as much as s/he wants.. When no tea packets are left, that game is over, thus subsequent participants cannot participate in the Resource Dilemma decision. The Resource Dilemma is last so that the participants won't know in advance.

Supplementary Reference

1. Johnstone, R. a & Cant, M. a. The evolution of menopause in cetaceans and humans: the role of demography. *Proc. Biol. Sci.* **277,** 3765–71 (2010).
